# Spontaneously implemented spatial coherence in vertical-cavity surface-emitting laser dot array

**DOI:** 10.1038/s41598-022-26257-0

**Published:** 2022-12-14

**Authors:** Tatsushi Hamaguchi, Tomohiro Makino, Kentaro Hayashi, Jared A. Kearns, Maho Ohara, Maiko Ito, Noriko Kobayashi, Shouetsu Nagane, Koichi Sato, Yuki Nakamura, Yukio Hoshina, Tatsurou Jyoukawa, Takumi Watanabe, Yuichiro Kikuchi, Eiji Nakayama, Rintaro Koda, Noriyuki Futagawa

**Affiliations:** Tokyo Laboratory 06, Sony Group Corporation, 4-14-1 Atsugi, Kanagawa, Japan

**Keywords:** Diode lasers, Semiconductor lasers

## Abstract

We report a self-induced spatially-coherent dot array consisting of fourteen units of vertical-cavity surface-emitting modes that exhibit spatially uniform spectra. A 47.5 µm total beam width and 0.5° narrow emission are achieved using an oblong cavity enclosed with a flat top mirror, cylindrically curved bottom mirror, and side facet. Notably, terminating the side of the cavity with a perpendicular facet enhances the horizontal propagation, which couples with the vertical resonance in each dot, similar to the case of master lasers in injection-locked lasers that delocalize the modes. Conventional semiconductor lasers, edge-emitting lasers, and vertical-cavity surface-emitting lasers have a Fabry–Pérot cavity; furthermore, emission and resonance are in identical directions, limiting the beam width to micrometers. Though the present structure has the same scheme of propagation, the right-angled facet synchronizes the modes and drastically expands the beam width.

## Introduction

The origin of lasers can be traced back to Einstein’s prediction of stimulated emission^1^, whereby population inversion allows lasing. Most semiconductor laser researchers believe that laser research will lead to better ways to confine carriers and light in a small area. Accordingly, widely used semiconductor lasers, such as edge-emitting lasers (EEL)^2,3^ and vertical-cavity surface-emitting lasers (VCSELs), have a high light confinement and small beam waists (approximately below ten microns). Consequently, these lasers have negligible power consumptions and are built inside industrial or consumer electronic devices as optical-disk drives, computer mice, laser printers, projectors, and so on^4^. However, this high confinement leads to wide emission angles due to diffraction^5^. In this context, these lasers require additional optical components to narrow the emission beam for many industrial applications, increasing the size and fabrication cost of the entire system. Thus, finding new cavity structures that allow narrow emissions at a small power consumption is of high interest.

Photonic-crystal surface-emitting lasers (PCSELs)^6^ are one of the candidates fulfilling the aforenoted requirements. The light in PCSELs propagates in the in-plane direction to attain resonance modulated by a periodic 2D-array of air holes of subwavelength sizes. The periodic holes direct the in-plane traveling photons in the vertical direction. This device allows sub-millimeter-wide beams with a very narrow emission angle of 0.1° and super-Watt-class outputs^7^. An alternative is injection-locked laser-arrays, wherein a single laser (the ‘‘master’’ laser) induces coherence among a collection of lasers (the “slave” lasers)^8,9^. This synchronizes the phase of VCSEL arrays to emit relatively narrow beams^10^. These arrays have shown extraordinary results by drastically changing the width of beams compared to that achieved using the conventional sigle-emitter lasers. However, such arrays entail several industrial challenges. For example, the mass fabrication of PCSELs is difficult owing to their subwavelength structures. The large area corresponding to current injection complicates heat management. Furthermore, injection-locked lasers encounter increased complexity with existence of two non-monolithic lasers.

A coherent array of self-coupled lasers is another potential solution to the aforementioned problem^11^. This approach creates coherence among lasers by constructing them close to each other. Such arrays have been intensively researched with VCSELs using optical coupling based on evanescent fields and diffracted light emitted from each laser unit. They can function without subwavelength structures or multiple non-monolithic lasers. Arranging emitters in a ring configuration widens the beam waist^12^, lowers the heat generation by emptying the inner part surrounded by these emitters, and narrows emissions. One remaining drawback of a self-induced coherent array is the complexity in establishing coupling. For example, the interval between the VCSELs must be carefully designed to couple efficiently by predicting the spatial distribution of evanescent fields. Additionally, setting a current level at which the emitters behave uniformly enough to attain coherence is difficult. Thus, controlling individual VCSELs is a challenge. At times, multiple wirings need to be provided to individual light emitters. However, increasing the number of emitters can exponentially deteriorate their control. Thus, the maximum number of VCSELs in an array is limited^13^, with a width of approximately 10 µm^12^.

**Figure 1:**
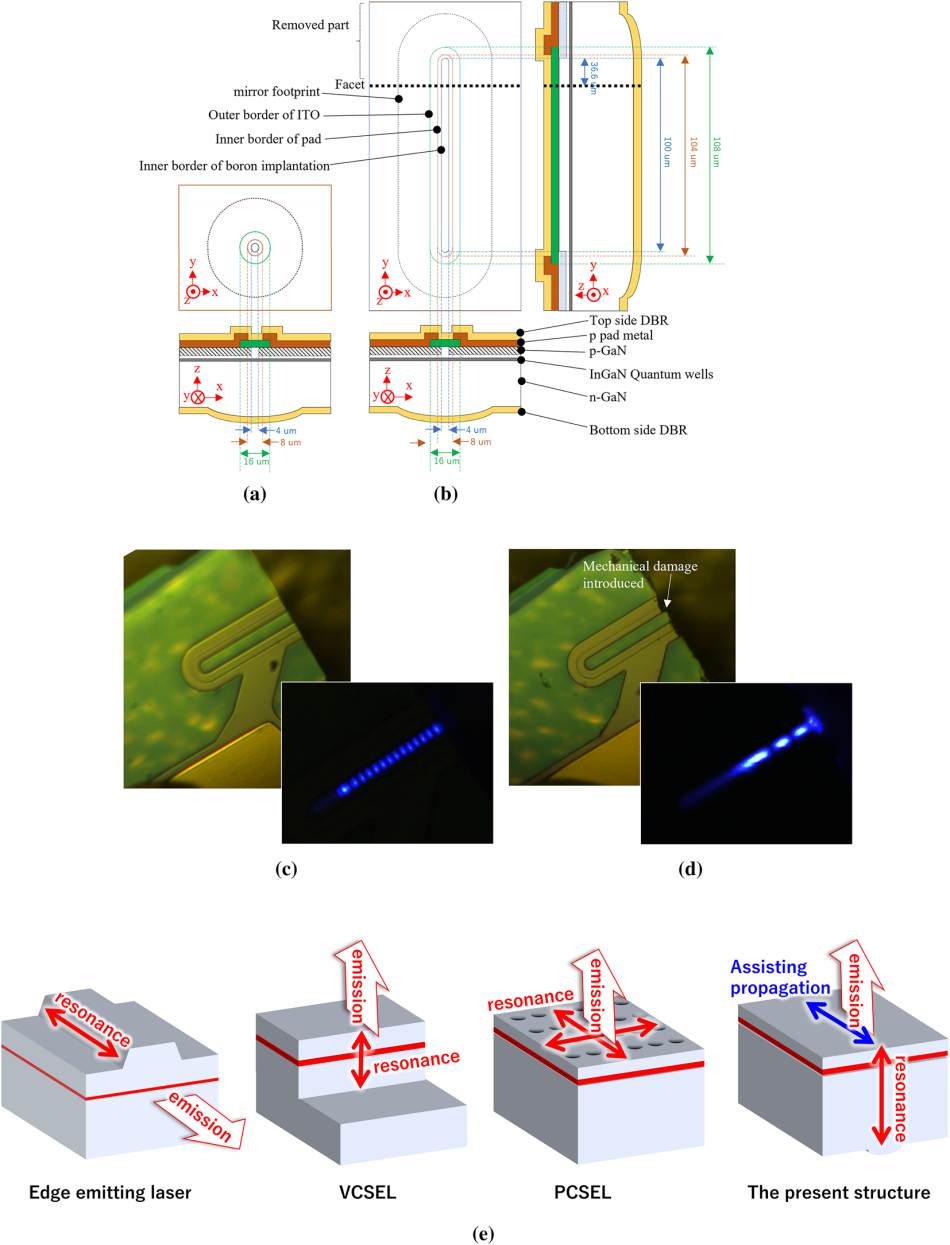
(**a**, **b**) show schematics of devices used for the present study. (**a**) shows the standard, referential, VCSELs with a curved mirror on one side. (**b**) shows the VCSEL with a cylindrical lens investigated in the present study. The distance between the top and bottom mirrors is 20–30 µm. The thickness of p-GaN is approximately 100 nm. InGaN multi quantum-well (MQW) is approximately 20 nm including the barrier layers. Thus, the rest of cavity is occupied by n-GaN. Top and bottom side Distributed Bragg reflector (DBR) are designed to have reflectivity of 99.7% and > 99.9%, respectively. Those devices are fabricated over (0001) plane of GaN substrate. The x-, y-, and z-directions of the GaN substrate are $\left\langle {{1}{-}{1}00} \right\rangle ,\left\langle {{11}{-}{2}0} \right\rangle ,{\text{ and}}\left\langle {000{1}} \right\rangle$, respectively. The fabricated devices have the structure shown in (**b**). Thus, a cleaved facet was introduced along the dashed line. (**c**, **d**) are the images of device shown in (**b**) with and without facet. Additional mechanical damage was introduced in the facet shown in (**d**) by the hitting a probe for measurement. These are recorded under pulsed current injection above the threshold current (RT, 1 µs, 0.1%, 17.7 k/cm^2^). (**e**) show schematics of conventional semiconductor lasers, edge emitting lasers, VCSELs, PCSELs and the present one.

This paper reports a self-induced spatially-coherent 1D dot-array containing 14 vertical-cavity surface-emitting modes spread over 47.5 µm using a unique cavity structure (see Fig. 1). It also contains a single rectangular aperture with a flat top and cylindrical bottom mirror. One end of the aperture is terminated with a perpendicular facet obtained by cleaving the device chip along the {11–20} plane of GaN.

## Results and discussion

**Figure 2:**
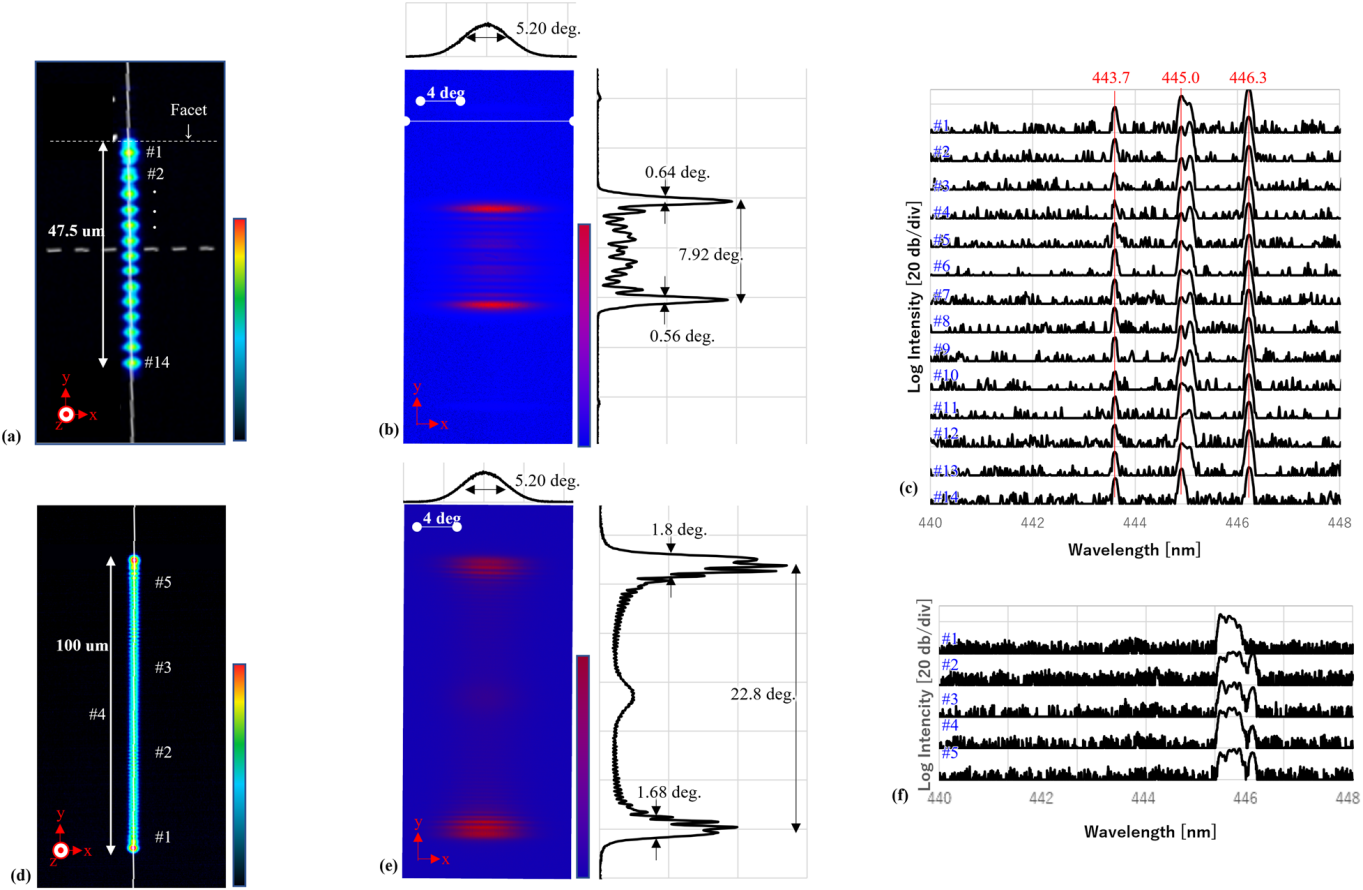
(**a**–**c**) are NFP, FFP, and spatial distributions, respectively, of the spectrum obtained for the device with a facet; (**d**–**f**), respectively, are those of the device without a facet. The vertical color spectra are intensity color axis in linear scale with arbitrary units, for (**a**–**d**).

**Figure 3:**
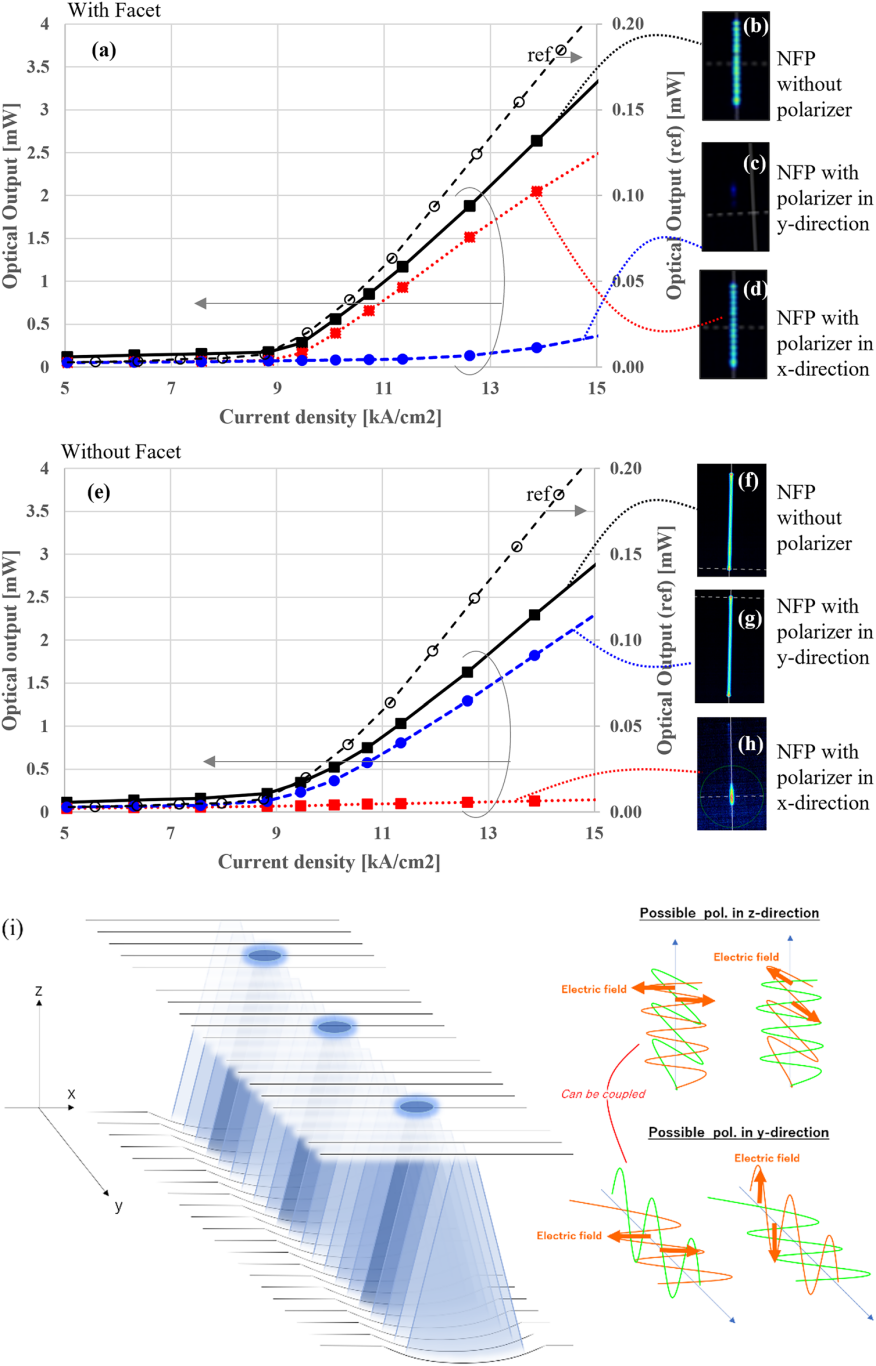
(**a**, **e**) are I–L curves obtained for the present devices with and without a facet, respectively. Each contains a VCSELs I–L curve for reference to show threshold. (**b**, **d**) are NFP images obtained just above the threshold current without and with a polarizer in x- and y-directions of a faceted device. (**f**, **h**) are those of the devices without facets. (**i**) is a schematic showing possible sets of polarization in the presently proposed cavity structure.

**Figure 4:**
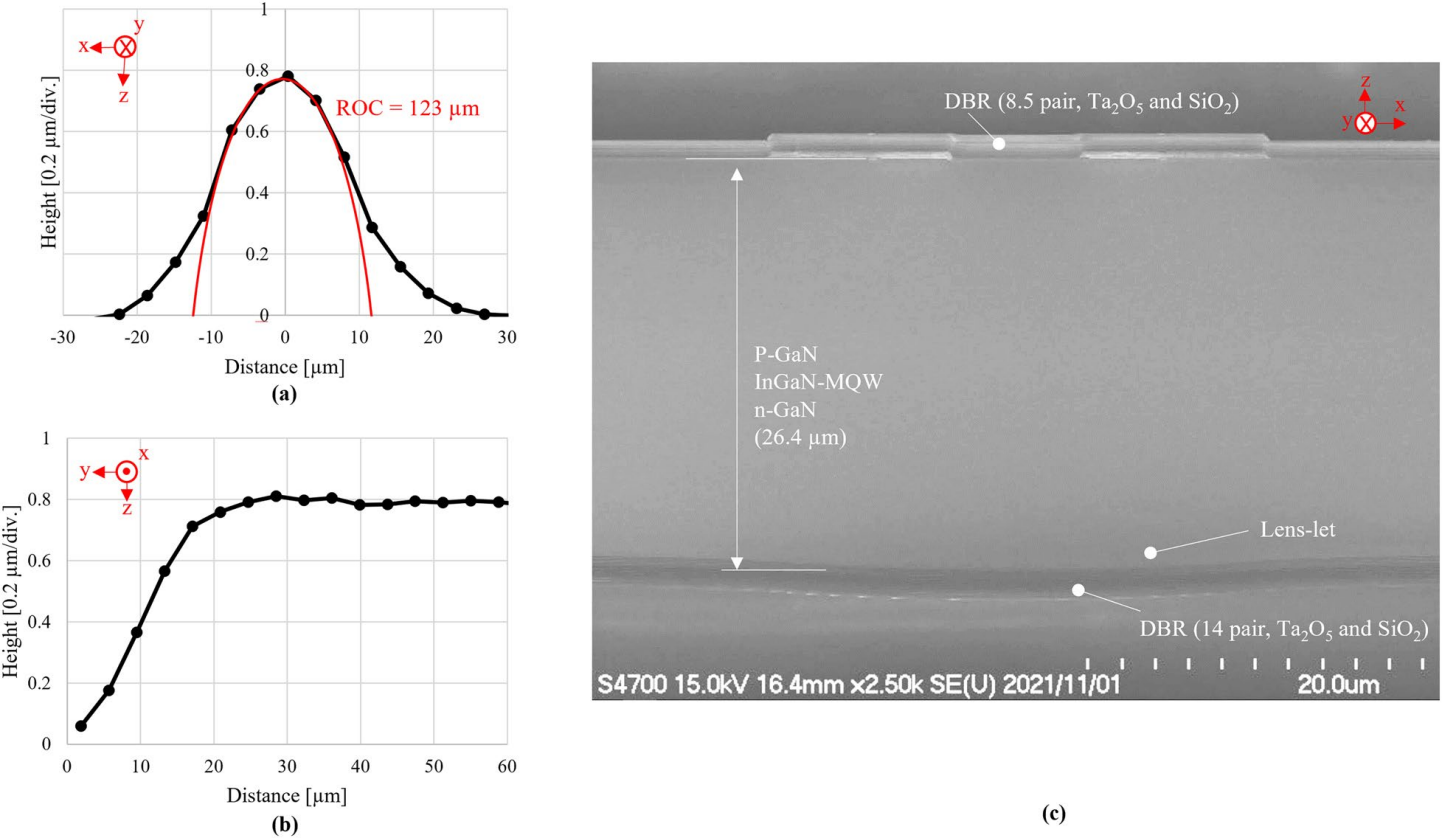
(**a**, **b**) are cross-sectional profiles of the cylindrical mirror obtained by laser-scanning confocal microscopy. (**c**) is cross-sectional SEM image obtained of the facet of the device and shows that the distance between two DBRs is 26.4 µm.

Figure 2A shows the near field pattern (NFP). The 14 dots are spread over 47.5 µm with an average interval of 3.7 um. Those dots filled the facet side of the 67-µm-wide oblong aperture. The full-width of half-maximum (FWHM) in the NFP in the x-direction was 2.8 µm. Figure 2b shows the device’s far field pattern, wherein there are two peaks with a FWHM of 0.64° and 0.56° in the y-direction and 5.2° in the x-direction. The two peaks occur on both sides of a 1D spatially-coherent array of VCSELs^11^. These patterns, dots in NFP and peaks in emission angle did not show any apparent shift during the measurement at any current level and any points of measurement given in this study. Figure 2c shows the spatial distribution of the spectrum observed for each dot. The positions of all peaks are stable across all 14 dots. The three peaks at 443.7, 445.0, and 446.3 nm have a 1.3 nm interval. It is equivalent to the longitudinal mode spacing estimated with a cavity length of 26.4 µm^19^. The cavity length was obtained through cross sectional analysis (see Fig. 4c). It indicates that the lasing mode has resonance in the vertical, i.e., z, direction. The black solid line in Fig. 3a is the J-V/L curve of the device with a facet. It showed a threshold current density (J_th_) of 9.0 kA/cm^2^. It indicates that these 14 dots act as a spatially-coherent 1D-array of vertical-cavity surface-emitting modes.

The delocalization is primarily caused by introducing the facet. Without the facet, the lateral mode did not produce coherent dots. It instead produced a featureless flat pattern in the NFP (seen in Fig. 2d), generating a wider far-field pattern (FFP (Fig. 2e) and a spectrum without any clear longitudinal modes (Fig. 2f). Even though some relatively sharp peaks in Fig. 2f exist close to ones in Fig. 2c, the broad peaks occupy the majority of observed optical intensity. Moreover, introducing some mechanical damage to one facet in the faceted samples disturbed the delocalization and left a localized chaotic pattern (seen in Fig. 1d). This further shows that the facet contributes to the delocalization of the mode. Figure 3a–h show the polarization behavior of devices with and without a facet. The directions of the electric fields in the facet device are perpendicular to the longitudinal direction (x-direction) of the aperture. Contrarily, the non-faceted device was polarized predominantly in the y-direction. Horizontal propagation did not entail polarization in the y-direction (perpendicular to the long side of the aperture). Thus, the vertical resonance in the devices without facets could never couple with horizontal propagation and vice versa. This further indicates that a facet could enhance the coupling between the vertical and horizontal propagations; please refer to Fig. 3i for a graphical understanding. This implies that the horizontal propagation acts as a “master” laser in injection-locked laser arrays to unite all “slave” lasers, i.e., the dots in this case, where horizontal propagation effectively entrains and synchronizes the phases of vertical resonance in the oblong aperture.

Cavities with parabolic curved and planar mirrors allow stable resonance by introducing lateral optical confinement^14^. The operation of VCSELs with such cavities has been reported^15^. The radius of curvature (ROC) is correlated to the width of the FFP of the device^16,17^. In the present study, the FWHM of the FFP in the direction corresponding to the short axis of the lens is 5.2°, which indicates that the ROC should be approximately 150 µm. It does not significantly influence the ROC value (123 µm) obtained through dimensional analysis (see Fig. 4a). This indicates that the light is confined by the curvature in the x-direction of the lens. It eliminates optical loss in this direction. However, the question of optical loss in the direction of the long axis of the lens, where only a flat top is observed (see Fig. 4b), remains unresolved. We created a standard VCSEL with a curved mirror^18–20^ as a reference for further consideration. This device had a curved mirror with a circular footprint instead of a cylindrical one (see Fig. 1a). The radius of curvature of the referential device (123 µm) was equal to that of the short direction of the cylindrical lens used in the present device. The J–L curve of this device appears in Fig. 3d as a dashed line. Remarkably, there is no apparent difference in the J_th_ of the present and referential devices. It indicates that the present cylindrical device does not suffer from increased optical loss in the long axis direction even if flat structures are present. Thus, the horizontal propagation to synchronize the modes has negligible optical power. Because the thresholds of disordered and ordered modes shown in Fig. 2a–e are consistent, the selection of modes due to facet introduction should not be caused by difference in the threshold. That further endorse the opinion that the horizontal propagation synchronizes transverse modes in the device with the facet through injection locking.

The present authors also consider similarity between the present device and slow-light waveguide amplifier^25^ where oblong aperture permits zig-zag light path and leaves dots with a constant interval in NFP. Considering the dots interval in the present device, 3.7 µm, and vertical cavity length, 26.4 µm, the zig-zag mode in the present device could provide dominant emission at ± 4 degree, which is consistent with the fact that the FFP shows intensive peak with interval of 7.92 degree (see Fig. 2b). The emission spectrum, shown in Fig. 1c showed three dominant peaks with the interval of 1.3 nm, which is finely consistent with the vertical cavity length, 26.4 µm as mentioned above. Remarkably, the center peak among these three has a blunt shoulder in the right side, which has an interval of 1.28 nm to the sharp peak in the right, which is equivalent to longitudinal mode spacing estimated for cavity length of 26.7 µm, slightly longer than the vertical cavity length of 26.4 µm. This analysis implies the zig-zag mode could play some role and be coupled with the dominant vertical modes.

Finally, the implications of the present study are considered. Conventional semiconductor lasers such as EEL and VCSELs have a Fabry–Pérot cavity wherein emission and resonation are in the same direction, limiting the beam width to micro-meters. The present structure has an identical scheme, wherein the amplifying resonance is parallel to the direction of emission. The horizontal propagation enhanced by the introduction of the side facet synchronizes the modes and drastically expands the beam width, e.g., 47.5 µm, to allow narrow emission in the range of 0.5°–0.6°; please refer to Fig. 1e for a graphical understanding. This structure could be obtained through simple processes and does not require complicated structures, unlike in the case of PCSELs and injection-lock lasers. This would be beneficial for practical use as a compact system for laser processing, long-distance lidars, communications, and illumination.

The cavity with curved and planar mirrors allows for stable resonance despite dimensional disturbances, such as tilt in the mirrors caused by mechanical stress. This is because the rotating-curved dimensions change the cavity structure negligibly. In the present device, the curvature may stabilize the cavity against deformations, such as unobservable minor twisting. Thus, though the array size is presently limited by the aperture length, increasing it may allow for larger spatially-coherent arrays. Moreover, adding highly reflective mirrors on the face might extend the length of dot-array. There remain rooms for future research. For example, investigating how the interval and numbers of dots are determined should be one topic with the research to this laser. As discussed, the horizontal propagation plays important role to stabilize the transverse behavior of the device. The authors believe that this extraordinary long vertical-cavity enabled this horizontal propagation. In conventional VCSELs, the cavity is short and filled with optical structures with varying refractive index profiles, which impede photons propagate horizontally.

Bending the aperture to form not an oblong but a ring and gaining a dimension-like half-sliced donut creates an annular cavity with a virtually circular-dot far-field pattern and a significantly narrow emission angle^21^. Such a cavity has been investigated with a meter-wide bulky setup and gives Bessel beams with a radial, or sometimes azimuthal, polarization. It is expected to produce a sharply focused spot rather than the more conventional linearly or circularly polarized beam^22^ and is used in optical trapping^23^. Applying such a half-donut structure to semiconductor lasers will potentially allow a large area for current injection, while avoiding heat generation center part of the ring which has caused heat management issues with PCSELs. This setup simultaneously gives a super-watt-class and narrow emission without heat management issues. Such a structure has ever not been achieved in monolithically fabricated semiconductor lasers.

An additional merit of the present device is the independent of choice of the material. The cavity structure is material independent as long as the cavity material is transparent to the resonating wavelength. Thus, this setup can employ families of semiconductors such as GaAs and InP, oxides such as perovskites and phosphors, and organic materials. This should further enable a variety of emission wavelengths for a plethora of applications.

## Methods

Figure 1A and b show the device structure used in the present study. The figure shows the detailed dimensional design of apertures, ITO layers, etc. The fabrication process was as follows. Metal–organic chemical-vapor deposition (MOCVD) was used to grow three quantum wells (InGaN/GaN MQWs), a p-GaN layer doped with Mg (~ 1 × 10^19^ cm^−3^), and a contact layer heavily doped with Mg (~ 1 × 10^20^ cm^−3^). They were grown to a total thickness of approximately 100 nm on a (0001) GaN substrate. A 30-nm-thick ITO layer and p-side DBR with 8.5 Ta_2_O_5_/SiO_2_ bilayers, with designed reflectivity about 99.6%^19^, were deposited on the contact layer via vacuum deposition under the same experimental conditions used in our previous studies^18–20^. A recess was etched adjacent to the aperture, exposing the n-GaN layer. Two Ti/Pt/Au electrodes were deposited to establish contact with the ITO layer and the exposed n-GaN, forming a current path. A current injection region was electrically confined via boron implantation^24^. The aperture was placed in contact with the ITO and Ti/Pt/Au electrodes. A wafer was lapped to a thickness of approximately 20–30 μm. Resin masks were photolithographed on the lapped face of the GaN wafer with an orientation of (000–1), as part of the lens-led fabrication process. By heating the specimen to 200 °C, the resin masks were melted into droplets for the referential VCSELs and cylindrical for the featured one. Reactive ion etching was used to transfer the surficial shape of the resin melts onto the GaN substrate by removing them as sacrificial masks, which transfer the similar shapes of melt resin onto the GaN. An n-side DBR with 14 Ta_2_O_5_/SiO_2_ bilayers, with designed reflectivity > 99.99%^19^, was deposited to form the curved and cylindrical mirrors. Finally, the device was diced and mounted on a ⌀9 TO-CAN package without sub-mounts in the p-up configuration.

The dimensional characterization of those deformed mirrors was realized via different methods before the deposition of the DBRs. The cross-sectional dimensions of the curved mirror were measured using confocal laser scanning microscopy (Keyence VK-X1000). The roughness of the top of the curved and plane surfaces formed on the GaN were measured using atomic force microscopy (AFM; Bruker Dimension Icon). We deposited DBRs with the same structure as that in the device on the BK7 glass plates as specimens to measure the reflectivity spectra of the DBRs. The reflectivity spectra of these samples were measured using a spectrophotometer (Hitachi U-4000).

**Figure 5:**
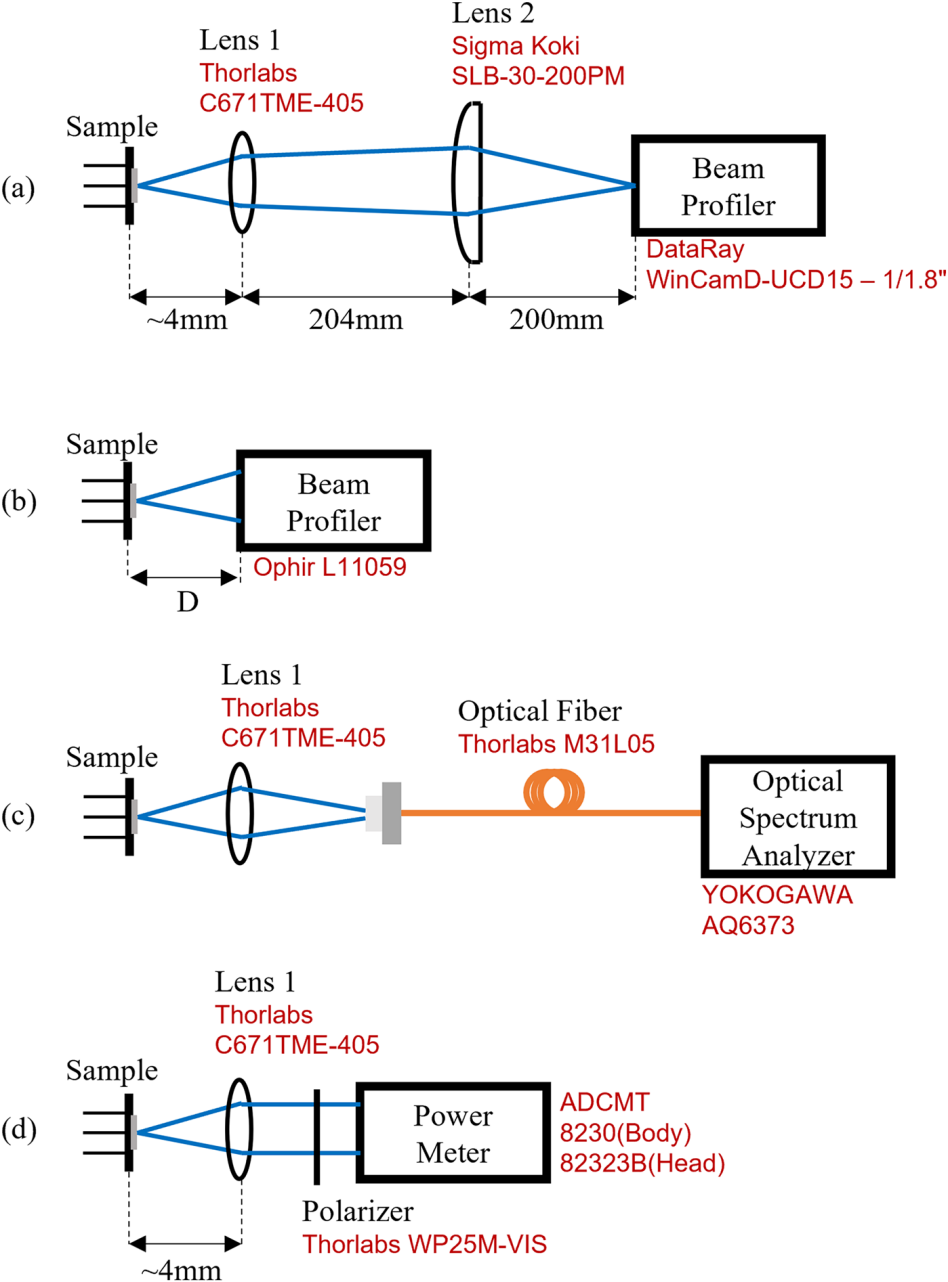
Setups used in the present study. (**a**–**d**) are for the measurement of NFP, FFP, spectrum, and I–V/L. The details are presented in the method section.

The measurements for the device were conducted with a specimen with a packaged finish in a 9φ TO-CAN. TO-CAN samples were fixed by a jig and driven at room temperature in the laboratory. In all measurements, a current source (ILXLightwave LDP-3811) was used to drive the VCSELs with a square-wave pulse signal with a duty ratio of 5% and pulse width of 1 µs. Figure 5a–d show the setups used for NFP, FFP, spectrum, and I-V/L measurements, respectively. The setup for the polarization measurement is shown in Fig. 5d, as a variation of I–V/L measurements. We used optical microscopy to record the image of the sample under current injection.

For NFP measurement (Fig. 5a), lens 1 (Thorlabs C671TME-405) and lens 2 (Sigma Koki SLB-30-200PM) are arranged such that the lens spacing equals the sum of their focal lengths. Accordingly, the system eliminates any astigmatism possibly caused by the oblong aperture. The image was captured on the beam profiler (DataRay WinCamD-UCD15-1/1.8").

In FFP measurement (Fig. 5b), a beam profiler (Ophir L11059) was used. The beam diameter and distance between the VCSEL and profiler are defined as d and D, respectively. The divergence angle of the FFP of emitted light was measured as θ = Tan^− 1(d/D).

Spatial distribution of Emission spectra (Fig. 5c) were measured by a spectrometer (Yokogawa AQ6373, resolution = 0.1 nm, sensitivity = HIGH1) using an optical fiber (Thorlabs M43L02 105 µm 0.22 NA). This setup gives a 50-times magnified image of NFP at the end of multimode optical fiber, which enable measurement of spatial distribution of spectrum by setting the location of fiber end. The connector of the fiber is attached to the stage and scanned to obtain special distributions (Fig. 2c,f).

For I–V and I–L measurements, the output light is collimated by lens 1 and then transmitted through the polarizer. Next, the light is injected into a power meter (ADCMT 8230 and 82323B) for optical power recording. The polarizer is mounted on a rotating holder that allows for control of the angle of the polarizer to investigate the polarization behavior of the device (Fig. 3a–e).

## Data Availability

All data generated or analyzed during this study are included in this published article.
